# High rate of HSV-1 reactivation in invasively ventilated COVID-19 patients: Immunological findings

**DOI:** 10.1371/journal.pone.0254129

**Published:** 2021-07-01

**Authors:** Jessica Seeßle, Theresa Hippchen, Paul Schnitzler, Julia Gsenger, Thomas Giese, Uta Merle

**Affiliations:** 1 Department of Gastroenterology, University Hospital Heidelberg, Heidelberg, Germany; 2 Institute of Virology, University Hospital Heidelberg, Heidelberg, Germany; 3 Institute of Immunology, University Hospital Heidelberg, Heidelberg, Germany; University of California Irvine, UNITED STATES

## Abstract

SARS-CoV-2 infection can lead to severe acute respiratory distress syndrome with the need of invasive ventilation. Pulmonary herpes simplex-1 (HSV-1) reactivation in invasively ventilated patients is a known phenomenon. To date very little is known about the frequency and the predisposing factors of HSV-1 reactivation in COVID-19. Therefore, we evaluated our cohort of invasively ventilated COVID-19 patients with severe pneumonia for HSV-1 in respiratory specimens and combined these results with functional immunomonitoring of the peripheral blood. Tracheal secretions and bronchial lavages were screened by PCR for HSV-1 positivity. Comprehensive immunophenotyping and quantitative gene expression analysis of Interferon-stimulated genes (IFI44L, MX1, RSAD2, ISIG15 and IFIT1) and IL-1 beta were performed in whole blood. Time course of infection beginning at symptom onset was grouped into three phases (“early” phase 1: day 1–10, “middle” phase 2: day 11–30 and “late” phase 3: day 31–40). Pulmonary HSV-1 reactivation was exclusively observed in the later phases 2 and 3 in 15 of 18 analyzed patients. By FACS analysis a significant increase in activated CD8 T cells (CD38^+^HLADR^+^) in phase 2 was found when compared with phase 1 (p<0.05). Expression of Interferon-stimulated genes (IFI44L, RSAD2 ISIG15, MX1, IFIT1) was significantly lower after HSV-1 detection than before. Taken together, reactivation of HSV-1 in the later phase of SARS-CoV-2- infection occurs in parallel with a drop of antiviral innate responsiveness as shown by decreased expression of Interferon-stimulated genes and a concurrent increase of highly activated CD38^+^HLADR^+^ CD8 T cells.

## Introduction

Coronavirus disease of 2019 (COVID-19), caused by infection with severe acute respiratory syndrome coronavirus 2 (SARS-CoV-2) causes a predominantly respiratory illness with a wide range of clinical severity, ranging from asymptomatic or mildly symptomatic to severe acute respiratory distress syndrome (ARDS) with the need of invasive ventilation [1–3].

Herpes simplex virus type 1 (HSV-1) infections are common in humans and replication can be detected in the distal airways of 5–64% of ICU patients receiving invasive ventilation [4, 5]. Mostly, HSV-1 reactivates in a state of reduced immunocompetence, but it is also reported in patients without immunosuppression [4]. Whether or not pulmonary HSV-1 reactivation causes morbidity or mortality in ICU patients is still controversially discussed [5–8]. Several case reports implicate a beneficial effect of aciclovir treatment in HSV-1 reactivation, but larger studies failed to confirm this observation [9, 10]. The decision for treatment should be based on the type of respiratory secretion positive for HSV-1, the viral load, and the clinical situation [11]. So far, only little is known about the frequency and the predisposing factors of HSV-1 reactivation in ventilated patients with COVID-19 pneumonia.

Acute viral infections are characterized by T cell activation. Both CD8 and CD4 T cells are required to clear an acute viral infection [12]. Additionally, interferon signaling as part of the innate immunity is important in preventing viral infections. Previous studies in severe COVID-19 patients demonstrated lymphopenia and dysregulated antiviral response by impaired type I/III IFN response [13, 14]. Recently it has also been shown that SARS-CoV-2-infection is associated with CD8 T cell activation in a subset of patients [15]. In this study especially the frequency of CD38^+^HLA-DR^+^ CD8 T cells was elevated in COVID-19 patients who had a concomitant infection with another pathogen but was not impacted by pre-existing immunosuppression or treatment with steroids [15].

To better understand the presence and frequency of HSV-1 reactivation in ventilated COVID-19 patients, we conducted a retrospective study of invasively ventilated COVID-19 patients with pneumonia who were admitted to our ICU. These patients were screened for pulmonary HSV-1 reactivation in tracheal secretions (TS) and bronchial lavages (BAL). In addition, FACS analysis of immune profile focused on T-lymphocytes and their subsets and gene expression analysis of Interferon-stimulated genes (ISGs) (IFI44L, MX1, RSAD2, ISIG15 and IFIT1) and IL1b by RT-PCR were done.

## Methods

### Study population

103 patients with laboratory confirmed SARS-CoV-2 infection with an age of 18 years or older that were followed as an inpatient at the University hospital of Heidelberg between March 18th and April 23rd, 2020 were considered for inclusion. Data analysis was approved (number S-148/2020) by the Ethics Committee of the Medical Faculty Heidelberg and was conducted in accordance with the Declaration of Helsinki. Written informed consent was obtained for all patients included in the study. Laboratory parameters were measured in the hospital central laboratory according to standard methods. Chest CT scan was done in 86.7% of the patients within 24 hours of admission to ICU. Chest CT scan was not repeated with the detection of pulmonary HSV-1 reactivation. None of the patients was treated with COVID-19-specific medication like dexamethasone or remdesivir during their hospital stay. A single dose of prednisolon was given in individual cases. Time course of infection beginning at symptom onset was grouped into three phases (“early” phase/phase 1: day 1–10, “middle” phase/phase 2: day 11–30 and “late” phase/phase 3: day 31–40). With the detection of HSV-1 in TS/BAL or serum antiviral treatment with aciclovir was started and continued until negative detection.

### Detection of HSV and SARS-CoV-2 in TS/BAL

Respiratory secretions (bronchoalveolar lavage fluid or tracheal aspirates) were tested for HSV-1/2 and SARS-CoV-2 by quantitative real-time PCR. Analytical sensitivity for each virus is presented in 1 000 copies/ml. In detail, DNA and RNA was isolated from bronchoalveolar lavage fluid or tracheal aspirates using QIAGEN kits (QIAGEN, Hilden, Germany) automated on the QIASymphony (DSP Virus/Pathogen mini Kits) and eluted in 115 μl elution buffer. For detection of HSV DNA and CMV DNA, RealStar kits (altona Diagnostics, Hamburg, Germany) were used according to the manufacturer’s recommendation. RT-PCR for SARS-CoV-2 was carried out using various reagent mixes–LightMix Modular SARS and Wuhan CoV E-gene, LightMix Modular SARS and Wuhan CoV N-gene, LightMix Modular Wuhan CoV RdRP-gene and LightMix Modular EAV RNA Extraction Control (as internal Control) from TIB MOLBIOL Syntheselabor GmbH (Berlin, Germany) and LightCycler Multiplex RNA Virus Master (Roche, Germany)–according to manufacturer’s instructions. RT-PCR was performed on LightCycler 480 or 480 II (Roche, Germany). Thermal profile is as follows: reverse transcription step at 55°C for 5 min, followed by denaturation at 95°C for 5 min, and 45 amplification cycles (denaturation at 95°C 5 sec, annealing at 60°C 15 sec, and elongation at 72°C for 15 sec).

### Immunophenotyping

The phenotyping was performed in whole blood within 4 hr. Absolute cell count was measured using the BDMultitest™ 6-color TBNK reagent with BD Trucount™tubes (BD Biosciences, Heidelberg, Germany). Employing 4 phenotyping panels, a comprehensive analysis of peripheral blood leukocytes was performed. The panels are modified recommendations of the “Human Immunophenotyping consortium” [16]. Briefly, Panel-1 consisted of 17 antibodies to characterize the major T-cell populations, including naïve and various memory/effector populations (CD2; CD3; CD4; CD8; TCR ƴδ; CD45RA; CD197 (CCR7); CD28; CD57 and CD127). Th1, Th2; Th17 and Tfh cells were identified with the following antibodies: CD183 (CXCR3); CD185 (CXCR5) and CD196 (CCR6). Activation was monitored with antibodies against HLA-DR, CD38, CD278 (ICOS) and CD279 (PD1). Using CD3 as a backbone for absolute quantification, with this comprehensive panel more than 200 defined subpopulations have been quantified in absolute numbers as well as in various ratios. Panel-2 consists of 8 parameters (CD3; CD19; CD20; anti-IgD; CD27; CD10; CD24 and CD38), allowing the identification of transitional, naïve and memory B-cells as well as circulating plasma blasts both in absolute numbers (CD19 backbone) and in various ratios. Panel-3 identified various NK, monocyte and DC subsets and included antibodies with the following specificities: CD11c; CD123; CD14; CD16; CD19; CD2; CD20; CD3; CD45; CD56; CD57; CD8a; HLA-DR; NKG2C and M-DC8. Using NK-cells as backbone, absolute numbers were calculated for 50 different populations. Finally, the Treg subset was characterized using the following antibodies: CD127; CD194 (CCR4); CD25; CD3; CD4; CD45; CD45RO and anti-HLA-DR. CD4+ T-lymphocytes served as backbone for the absolute quantification of the different subsets. The analysis was performed on an LSR Fortessa Analyzer (Special Order Research Product) (BD Biosciences).The detailed setup, staining, gating and analysis procedures have been recently described [17].

### Gene expression analysis

0.5 ml of heparinized whole blood was incubated with 0.1 ml RPMI1640 for 3 hours at 37°C and 7% CO_2_. Red cells were lysed with ACK-buffer 2-times. Afterwards, the leukocytes were lysed with 400 μl of MagNA-Pure lysis buffer (Roche) containing 1% DTT ((1,4-Dithiothreitol, Roche Cat. #: 10 708 984 001)) and the samples were frozen at –70°C. After thawing, the lysates were well mixed and transferred into the MagNA-Pure samples cartridge and mRNA was isolated with the MagNA-Pure-LC device using the mRNA standard protocol for cells. The elution volume was set to 50μl. An aliquot of 8.2 μl mRNA was reversely transcribed using AMV-RT and oligo- (dT) as primer (First Strand cDNA synthesis kit, Roche) according to the manufactures protocol in a thermocycler. After termination of the cDNA synthesis, the reaction mix was diluted to a final volume of 500 μl and stored at –20˚C until PCR analysis. The following primer sets optimized for the LightCycler® (RAS, Mannheim Germany) were developed and purchased from SEARCH-LC GmbH (www.Search-LC.com):

IFI44L_3:GCGTCAAAATAAATTAATTGTAGACCT;IFI44L_5:TTGTCTCCATACAGTTGGGTTG;

IFIT1_3:TTTTGTAGCCTCCTTGATTTGGAT; IFIT1_5:CATTGAAGAAGCTCTAGCCAACA;

IL1B_5:CCTGTACGATCACTGAACTGCACGC;IL1B_3:GCTTATCATCTTTCAACACGCAGGA;

ISG15_3:CTGCGGCCCTTGTTATTCCT;ISG15_5:ATGGGCTGGGACCTGACGGTGAAG;

MX1_3:CCGGCACTTGACAATCATGTAACC:MX1_5:CCCGGATCTGACTCTAATAGACCT;

PPIB_3:GAAGCGCTCACCGTAGATGC;PPIB_5:GCCCAAAGTCACCGTCAAGG;

RSAD2_3:AGCTTCAGATCAGCCTTACTCCA;RSAD2_5:AGAGAAGCAGAAAGATTTGTTATTGGT The PCR was performed with the LightCycler® FastStart DNA Sybr GreenI kit (RAS) according to the protocol provided in the parameter specific kits. To control for specificity of the amplification products, a melting curve analysis was performed. The absolute copy number was calculated from an external standard curve, obtained by plotting known input concentrations of four different plasmids at log dilutions to the PCR-cycle number (CP) at which the detected fluorescence intensity reaches a fixed value. To correct for differences in the content of RNA, the calculated transcript numbers were normalized according to the expression of the housekeeping gene peptidylprolyl isomerase B (PPIB). Values were thus given as transcripts per 1000 transcripts of PPIB.

### Statistical analysis

Variables were described by median (interquartile range, IQR) or absolute (relative, %) frequencies for metric or categorical variables, respectively. Classifications with respect to limits of normal (above and/or below limits) for laboratory findings are described by absolute (relative, %) frequencies. Comparison between phase 1, 2 and 3 was performed by Wilcoxon signed-rank test for metric or variables and restricted to patients with both measurement times observed. Descriptive p-values are reported and p-values<0.05 considered statistically significant. Analyses were performed with IBM SPSS Statistics for Windows, Version 24.0, Armonk, NY: IBM Corp.).

## Results

### Patient characteristics and laboratory findings

**Fig 1:**
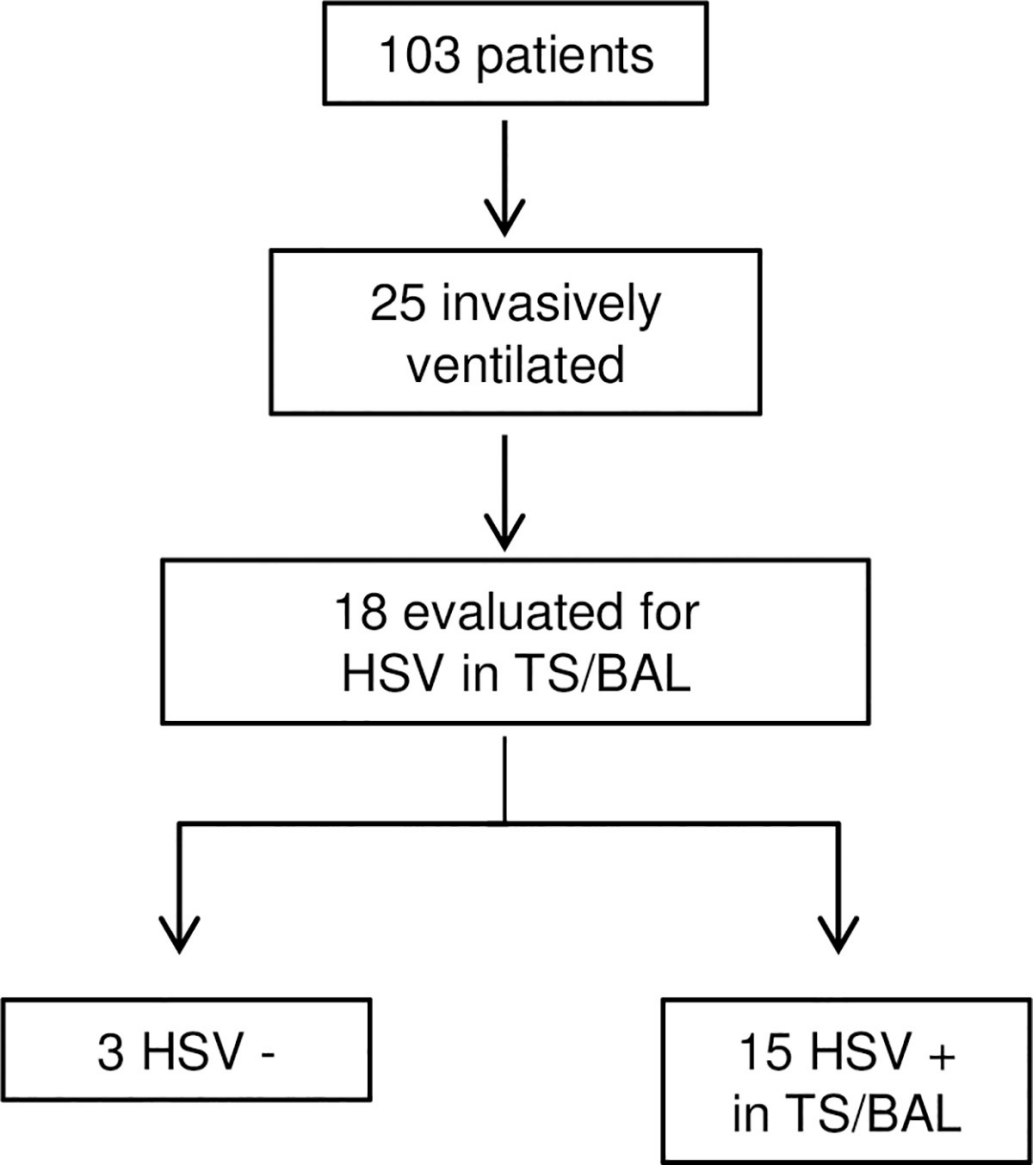
Flow chart of study population and HSV-1 detection in Tracheal Secretion (TS) or bronchial lavages (BAL).

The study population with confirmed diagnosis of SARS-CoV-2 infection is represented in Fig 1. Of 103 hospitalized patients 25 patients (24.3%) were invasively ventilated because of severe or critical pneumonia. For 18 of 25 invasively ventilated patients respiratory specimens for further analysis were available and analyzed for HSV-1 reactivation by PCR in tracheal secretions (TS) or bronchial lavages (BAL). In 15 of these 18 patients HSV-1 was detectable (83.3%). All patients with HSV-1 detection had positive HSV-1 IgG in serum with a median of 222 (IQR 64) g/l. No HSV-1 DNA was detectable in samples from 3 patients (16.7%, Table 1). The characteristics of the 15 positively tested patients are outlined in Table 1. 80.0% of the patients were male and 86.7% had a COVID-19-typical chest CT-scan at admission. All patients received invasive ventilation because of ARDS. ARDS stage was in 73.3% of the patients mild, in 20.0% moderate and in 6.7% severe, respectively. Median duration of invasive ventilation was 8.5 (IQR 9.3) days. 40.0% of patients received renal replacement therapy. Bacterial superinfection was observed in 26.7%. Comorbidities in COVID-19 patients were mostly cardiovascular (66.7%). 33.3% had a diabetes mellitus type 2. 6.7% and 13.3% patients had a known diagnosis of cancer or solid organ transplantation, respectively.

**Table 1:** Demographics of study population at first HSV-1 detection in TS or BAL.

	COVID-19
	n = 15
	n (%) or median (IQR)
Age (years)	71.0 (16.0)
Gender (male)	12 (80.0%)
Temperature (°C)	38.1 (1.2)
Preexisting conditions	
Cardiovascular	10 (66.7%)
Pulmonal	0 (0%)
diabetes mellitus type II	5 (33.3%)
Malignancy	1 (6.7%)
solid organ transplantation	2 (13.3%)
COVID-19-typical chest CT scan	13 (86.7%)
Invasive ventilation (d)	8.5 (9.3)
Oxygen support category	
Non-invasive ventilation via high-flow nasal oxygen	4 (26.7%)
Invasive ventilation	11 (73.3%)
Severity of ARDS	
Severe	1 (6.7%)
Moderate	3 (20.0%)
Mild	11 (73.3%)
Hospital stay (d)	13.0 (10.5)
Renal replacement therapy	6 (40.0%)
Bacterial superinfection	4 (26.7%)
HSV-1 DNA in respiratory specimen	
tracheal secretion	9 (60.0%)
bronchial lavage	6 (40.0%)
HSV-1 DNA in serum	1 (7.7%)
HSV-1 IgG	15 (100%)
HSV-1 IgG (g/l)	222.0 (64)

Median (IQR) or frequencies n (%) are shown for the group characterization.

ARDS, Acute respiratory distress syndrome; BAL, bronchial lavage; CMV, cytomegalovirus; COVID-19, corona virus disease-19. HSV-1, Herpes simplex virus 1; TS, tracheal secretion.

Laboratory findings at first HSV-1 detection after symptom onset are summarized in Table 2. As in previous studies analyzing COVID-19 patients, we observed an inflammatory syndrome in our cohort. Median procalcitonin (PCT) and C reactive protein (CRP) were notably increased and outside the limits of normal in all patients. Ferritin was elevated in 10 patients (66.7%). Interleukin-6 (IL-6) was normal in 1 patient (6.7%), moderately (6–20 pg/ml) in 1 patient (6.7%), and highly (>21 pg/ml) elevated in 13 patients (86.7%). Absolute lymphopenia was present in 10 patients (66.7%).

**Table 2:** Laboratory data in median (IQR) of study population.

	Limits of normal	COVID-19	Outside the limits of normal (%)
		n = 15	
		median (IQR)	
WBC	4–10 /nl	11.0 (8.2)	60%
Lymphocytes	1.0–4.8 /nl	0.9 (0.8)	66.6%
Hemoglobin	13–17 g/dl	9.9 (2.0)	100%
Platelets	150–440 /nl	278.0 (102.0)	13.3%
Creatinine	0.6–1.2 mg/dl	2.0 (3.6)	80%
ALT	<50 U/l	47.0 (33.0)	40%
LDH	<317 U/l	452.0 (167.0)	86.7%
CRP	<5 mg/l	87.0 (165.5)	100%
PCT	<0.05 ng/ml	0.8 (2.3)	100%
Ferritin	<300 μg/l	752.0 (1412.0)	66.7%
PT (quick)	70–125%	70.2 (19.7)	40%
D-dimer	<0.5 mg/l	4.3 (8.0)	100%
IL-6	>6 pg/ml	65.7 (124.2)	93.3%
pro-BNP	<125 ng/l	1357.0 (7487.0)	100%
TNT (high sensitive)	<14 pg/ml	40.0 (153.0)	93.3%

WBC, white blood cells; ALT, alanine aminotransferase; LDH, lactate dehydrogenase; CRP, c reactive protein; PCT, procalcitonin; PT, prothrombin time; IL-6, interleukin-6; pro-BNP, B-type natriuretic peptide, TNT, troponin T.

### Course of HSV-1 and SARS-CoV-2 detection in TS/BAL and corresponding immune phenotyping

**Fig 2:**
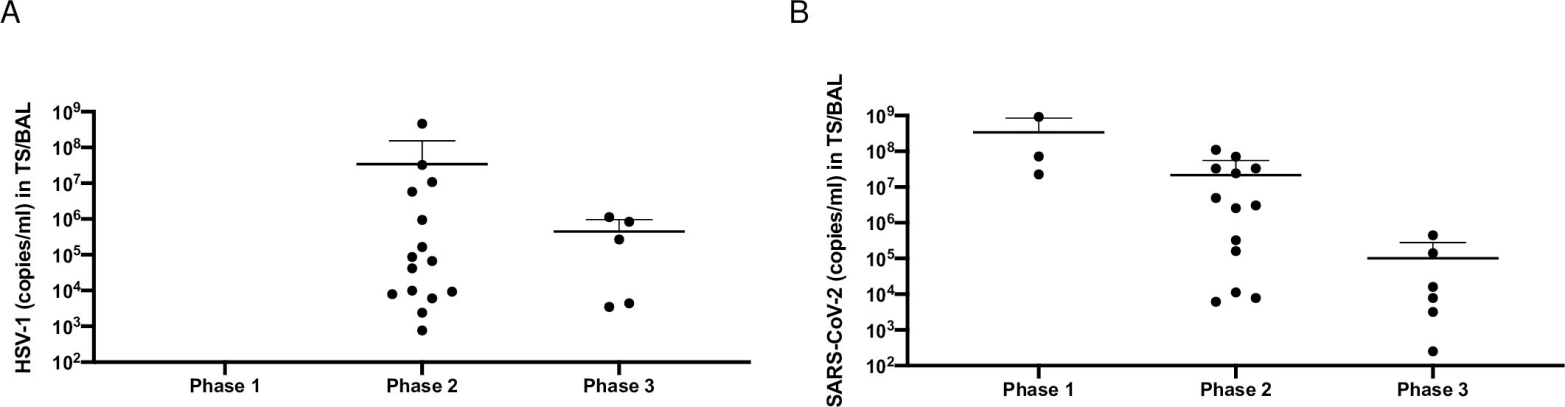
Time course of HSV-1 *(A)* and SARS-CoV-2 *(B)* detection in tracheal secretion (TS) or bronchial lavages (BAL) in copies/ml represented in 3 phases (phase 1: day 1–10, phase 2: day 11–30 and phase 3: day 31–40) starting at symptom onset.

**Fig 3:**
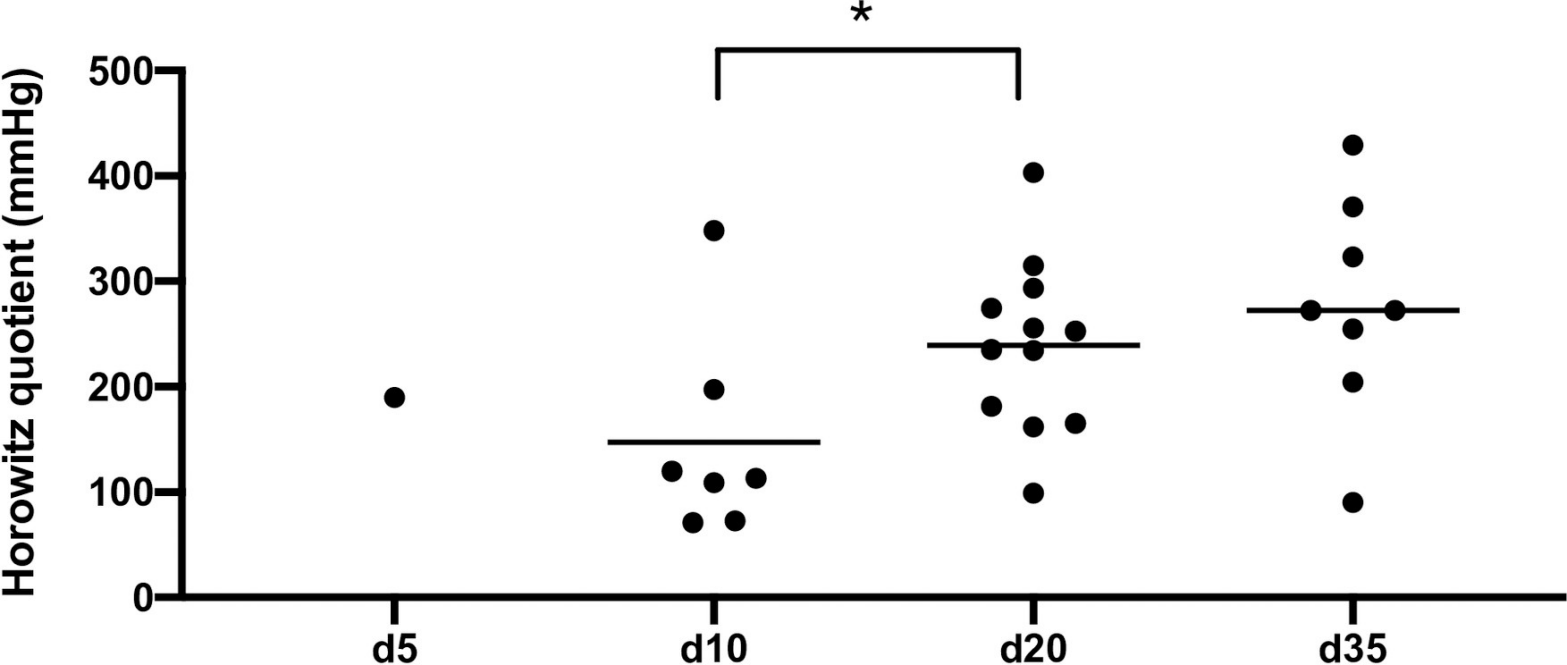
Horowitz quotient calculated at day 5, 10, 20 and 35 after symptom onset with a significant lower Horowitz quotient at day 10 in comparison to day 20 and 35. (*p<0.05).

15 invasively ventilated COVID-19 patients with HSV-1 reactivation in TS or BAL were further analyzed. First HSV-1 detection after symptom onset is outlined in Table 3. Time course of infection starting at symptom onset is grouped into 3 phases (“early” phase/phase 1: day 1–10, “middle” phase/phase 2: day 11–30 and “late” phase/phase 3: day 31–40). Fig 2 represents the course of viral load (copies/ml) in TS or BAL of HSV-1 (A) and SARS-CoV-2 (B). In phase 1 no HSV-1 reactivation was detected, followed by an obvious increase in phase 2 and 3. The Horowitz quotient was calculated at day 5, 10, 20 and 35 after symptom onset (Fig 3). The Horovitz quotient is the ratio of the arterial oxygen partial pressure (paO2) to the inspiratory oxygen concentration (FiO2). The severity of ARDS is classified according to the Berlin classification from 2012 using the Horovitz quotient: mild ARDS: 300–201 mmHg; moderate ARDS: 200–101 mmHg, severe ARDS: ≤100 mmHg. The median Horowitz quotient at day 10, 20 and 35 was 113.2 mmHg, 243.8 mmHg and 272.3 mmHg, respectively. At day 10 a significant lower Horowitz quotient was measured when compared with day 20 or 35 (day 10 vs. 20). No difference was observed between day 20 and 35.

**Table 3:** HSV-1 testing in TS/BAL of study population.

Patient No.	First HSV-1 detection after symptom onset (days)	Negative HSV-1 testing before first HSV-1 detection
1	12	✓
2	18	✓
3	18	✓
4	13	✓
5	18	✓
6	22	✓
7	17	✓
8	14	✓
9	17	Not done
10	28	Not done
11	31	Not done
12	27	Not done
13	19	Not done
14	23	Not done
15	15	Not done

**Fig 4:**
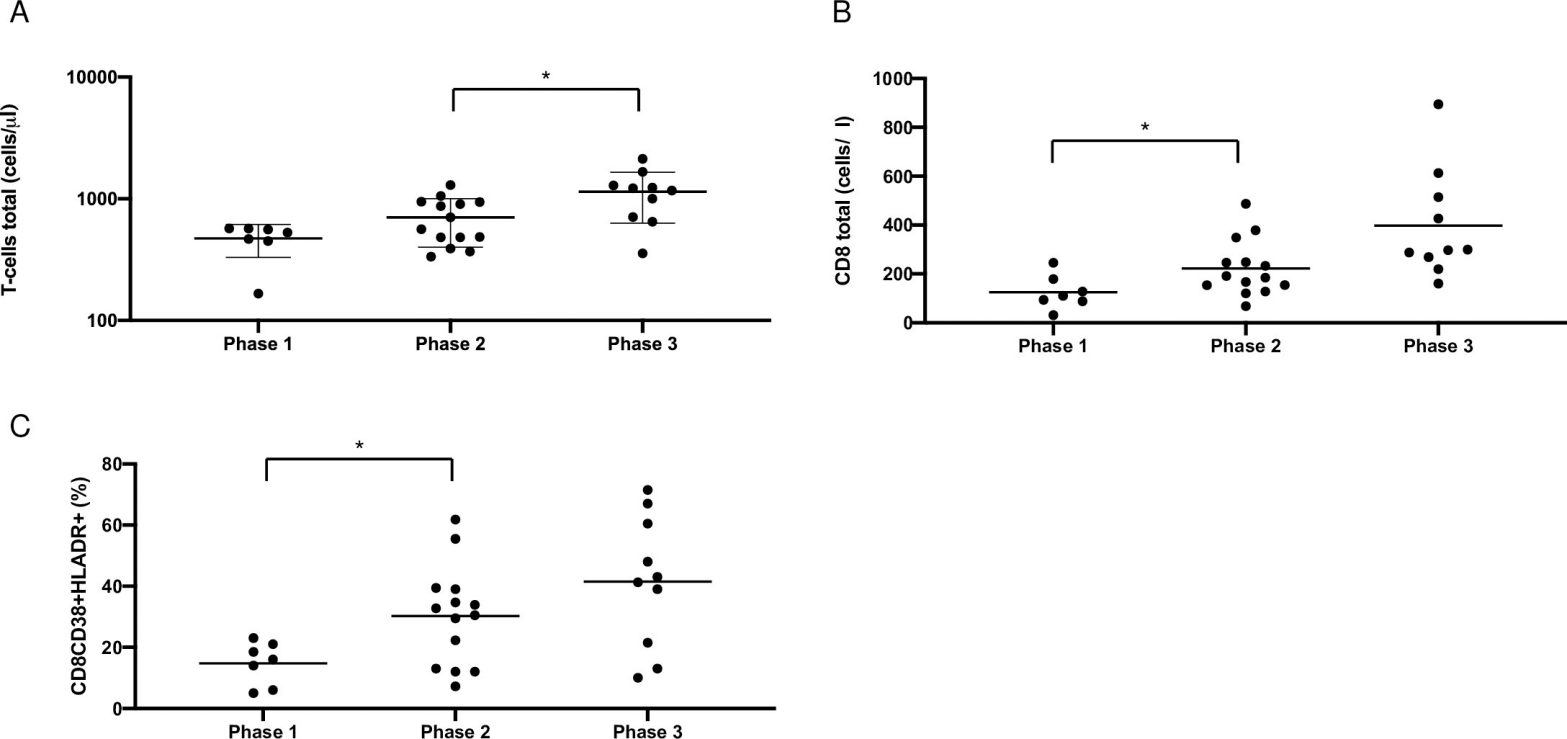
Total T cells (copies/μl) count *(A)*, total CD8 T cells (copies/μl) *(B)* and CD8CD38+HLADR+ T cells (%) *(C)* of COVID-19 patients with pulmonary HSV-1 reactivation measured in peripheral blood represented in 3 phases (phase 1: day 1–10, phase 2: day 11–30 and phase 3: day 31–40) starting at symptom onset (*p<0.05).

**Fig 5:**
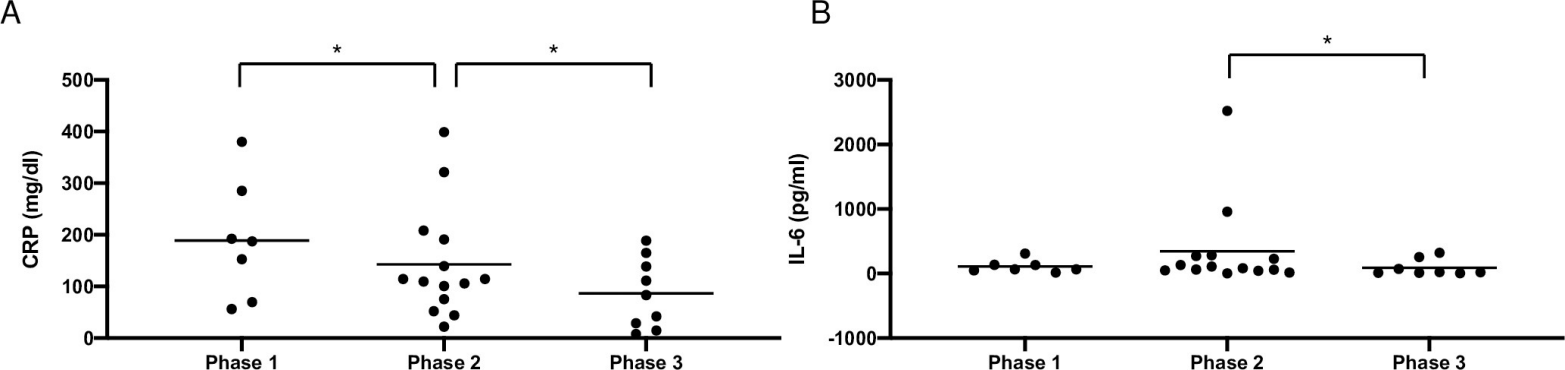
C reactive protein (CRP) *(A)* and interleukin-6 (IL-6) *(B)* of peripheral blood of COVID-19 patients with pulmonary HSV-1 reactivation represented in 3 phases (phase 1: day 1–10, phase 2: day 11–30 and phase 3: day 31–40) starting at symptom onset. (*p<0.05).

To further analyze immune response, comprehensive immunophenotyping of peripheral blood was performed. A significant increase of total T cells (phase 2 vs. 3, p<0.05), total CD8 T cells (phase 1 vs. 2, p<0.05) and activated CD8 T-cells (CD38^+^HLA-DR^+^) (phase 1 vs. 2, p<0.05) (Fig 4A–4C) was present. CRP was significantly decreased between phase 1 and 2 (p<0.05) and IL-6 between phase 2 and 3 (p<0.05, Fig 5A and 5B).

### Interferon-stimulated gene expression

**Fig 6:**
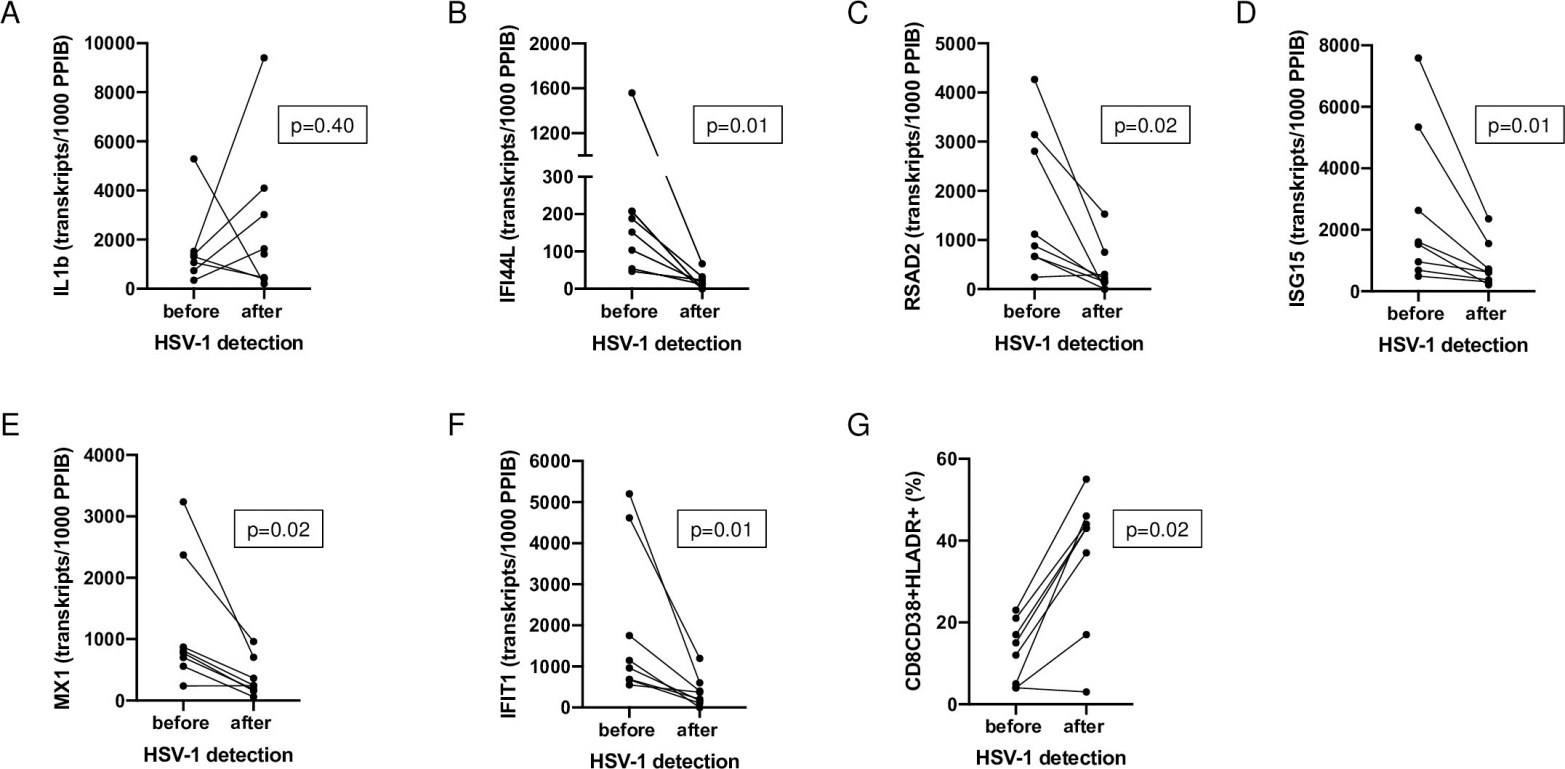
IL1b (A), Interferon-stimulated gene expression of IF44L (B), RSAD2 (C), ISG15 (D), MX1 (E) and IFIT1 (F) and CD8CD38+HLADR+ T cells (%) (G) of COVID-19 patients with pulmonary HSV-1 reactivation represented before and after HSV-1 detection in respiratory specimens.

**Fig 7:**
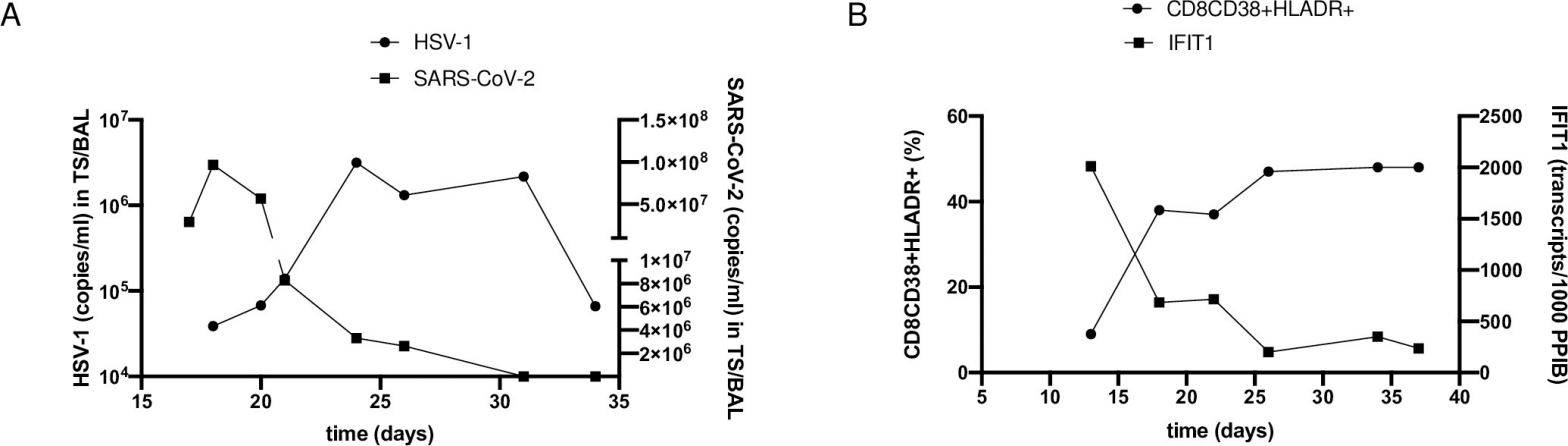
Time course of viral load of HSV-1 (copies/ml) and SARS-CoV-2 (copies/ml) in TS/BAL *(A)*, CD8CD38+HLADR+ (%) and IFIT1 (transcripts/1000 PPIB) *(B)* exemplarily illustrated for one patient.

The expression of the interferon-stimulated genes (ISGs): IFI44L, MX1, RSAD2, ISIG15, and IFIT1 as well as IL-1b in peripheral blood was analyzed and shown as transcripts/1000 transcripts of the PPIB gene (Fig 6A–6F). The ISGs and additionally activated CD8 T-cells (CD38+HLA-DR+) are presented before and after HSV-1 detection in respiratory specimens. A significant decrease was observed for FI44L, MX1, RSAD2, ISIG15, and IFIT1 (Fig 6B–6G) indicating a dysregulated type I/III interferon response in COVID-19 patients with pneumonia with a downregulation of ISGs. Exemplary, the time course of viral load of HSV-1 and SARS-CoV-2 in TS/BAL, CD38^+^ HLA-DR^+^ CD8 T cells and IFIT1 has been illustrated for one patient (Fig 7A and 7B).

## Discussion

The results of our retrospective analysis show that HSV-1 DNA was detected in 83% of invasively ventilated COVID-19 patients analyzed for HSV-1 in lower respiratory specimens in our study cohort. This is a higher rate of HSV-1 detection than previously reported rates for ventilated Non-COVID-19 patients ranging between 5 and 64% [18–20]. HSV-1 was detected after a median time of 9 days of mechanical ventilation. HSV-1 reactivation occurs in the later disease phases and is not present from the beginning as 8 of the 15 patients were negatively tested in BAL or TS. These results indicate that COVID-19 patients are more susceptible to HSV-1 reactivation may be due to a dysregulated host immune response as previously described and especially after longer periods of critical illness and mechanical ventilation [21–23]. Factors impairing the anti-viral immune control in COVID-19 patients are the partly severe lymphopenia and the impaired innate interferon response cause by SARS-CoV-2 [24].

Indeed, HSV-1 reactivation parallels the decrease of Interferon-stimulated gene (ISG) expression. Importantly, this drop was not observed in the three patients without HSV-reactivation. Interferons are classified as type-I, -II, or -III [25]. Type I interferons (IFNs) are key components in the antiviral response and inhibit viral replication through type-I IFN receptor (IFNAR) signaling and initiate transcription of hundreds of ISGs that have antiviral, immune modulatory, and cell regulatory functions [26]. As previously described the host response to SARS-CoV-2 induces a dysregulated IFN-I and -III response with minimal concentrations of type I interferons in peripheral blood of COVID-19 patients [21–23]. Low levels of IFN seem to correlate with disease severity [24]. *In vivo* and *in vitro* studies have revealed for HSV-1 and -2 infections several mechanisms to interfere with the host IFN response at multiple levels inducing impaired IFN secretion and related signaling pathways [27–29]. In our study we observed a significant lower expression of ISGs (IFI44L, RSAD2 ISIG15, MX1, IFIT1) after HSV-1 detection than before.

In parallel to the drop of ISG expression and HSV-1 reactivation, we observed an increasingly high percentage of activated CD8+ T cells displaying high and persistent expression of the CD38^+^ HLA-DR^+^ activation markers. All of the COVID-19 patients with HSV-1 reactivation had an increased amount of these activated CD8 T cell subset. Recently, it has been shown that the frequency of CD38^+^ HLA-DR^+^ CD8 T cells was elevated in COVID-19 patients who had a concomitant infection with other pathogens but were not impacted by pre-existing immunosuppression or treatment with steroids [15]. Of interest, in this study 20% of patients had no increase in CD38^+^ HLA-DR^+^ CD8 T cells above the level found in healthy donors, whereas in our cohort with HSV-1 reactivation all patients showed an increase in this cell population [15]. Additionally, it has been demonstrated that these changes in CD8 T cell subsets in COVID-19 patients did not show clear correlations with individual metrics of clinical disease such as hsCRP or d-dimer [15]. Of note, in our cohort CRP levels at first HSV-1 detection had already declined and the individual T cell count increased when compared to the levels of the initial phase. Therefore, the increase in activated cytotoxic T cells can be postulated as a correlate for developing an anti-viral adaptive immune response. However, as the occurrence of these cells is associated also with other infectious and non-infectious events activating T cells, this phenomenon is not specific for HSV-1. If the HSV-1 reactivation in respiratory specimen aggravates the dysregulated immune response or is due the acute SARS-CoV-2 infection remains unclear. Further studies have to follow to clarify this aspect. The limitation of our study is the small size, the retrospective approach, and that we did not differentiate between asymptomatic carriage and/or local HSV-1 detection and true infection (lung parenchymal involvement), as defined by HSV-1 detection and specific herpetic nuclear inclusions in the tracheal secretions.

## Conclusions

The detection of HSV-1 in the later phase of SARS-CoV-2- infection occurs in parallel with an increase in activated CD38^+^HLADR^+^ CD8 T-cells and a decreased expression of Interferon-stimulated genes indicating a dysregulated immune response. Whether HSV-1 reactivation has an impact on disease severity of ventilated COVID-19 patients and whether patients benefit from HSV-1 antiviral therapy has to be analysed in future studies.

## Supporting information

S1 Data (DOCX)
