# Supplementary material for: High rate of HSV-1 reactivation in invasively ventilated COVID-19 patients: Immunological findings
Source: PLoS One. 2021 Jul 1;16(7):e0254129. doi: 10.1371/journal.pone.0254129 (PMC8248692; doi:10.1371/journal.pone.0254129)
Supplement: S1 Data — (DOCX) [file pone.0254129.s001.docx]

| **Pat. No.:** | **1** | **2** | | **3** | **4** | **5** | **6** | **7** | **8** | **9** | **10** | **11** | **12** | **13** | **14** | **15** |
| --- | --- | --- | --- | --- | --- | --- | --- | --- | --- | --- | --- | --- | --- | --- | --- | --- |
| Age (years) | 78 | 77 | | 79 | 53 | 58 | 71 | 59 | 80 | 62 | 76 | 71 | 64 | 62 | 72 | 67 |
| Gender (m/f) | m | f | | m | m | m | m | m | m | f | m | m | m | m | m | w |
| **Preexisting disease** | | | | | | | | | | | | | | | | |
| - cardial |  | x | | x |  |  | x | x | x | x | x | x |  | x | x |  |
| - pulmonal |  |  | |  |  |  |  |  |  |  |  |  |  |  |  |  |
| - D.m. type II |  |  | | o |  |  | x | x |  |  | x | x | x |  |  |  |
| - malignancy |  |  | |  |  |  |  |  |  |  |  |  | x |  |  |  |
| Solid organ transplantation |  |  | |  |  | x |  |  |  |  |  |  | x |  |  | x |
| COVID-typical chest pain | x | x | | x | x | x | x | x | x | x | x | x | x |  | x |  |
| Hospital stay (days) | 8 | 20 | | 11 | 9 | 2 | 15 | 20 | 8 | 18 | 9 | 19 | 15 | 12 | 22 | 9 |
| Invasive ventilation (days) | 8 | 19 | | 7 | 6 | 2 | 10 | 12 | 8 | 8 | 8 | 19 | 13 | 5 | 12 | 9 |
| Renal replacement therapy | x |  | |  |  |  |  |  |  |  | x | x | x |  | x | x |
| **ARDS** | | | | | | | | | | | | | | | | |
| - severe |  |  | | x |  |  |  |  |  |  |  |  |  |  |  |  |
| - moderate |  |  | |  |  |  | x |  | x |  | x |  |  |  |  |  |
| - mild | x | x | |  | x | x |  | x |  | x |  | x | x | x | x | x |
| Bacterial superinfection |  | x | |  |  |  |  | x |  |  |  | x |  |  | x |  |
| **HSV** | | | | | | | | | | | | | | | | |
| - tracheal secretion |  | x | | x | x |  |  |  | x | x | x | x | x | x |  |  |
| - BAL | x |  | |  |  | x | x | x |  |  |  |  |  |  | x | x |
| **Laboratory findings** | | | | | | | | | | | | | | | | |
| WBC (/nl) | 18,6 | | 11,0 | 9,4 | 12,3 | 12,1 | 7,1 | 17,8 | 8,8 | 6,8 | 5,1 | 10,6 | 6,6 | 13,5 | 15,3 | 25,7 |
| Lymphocytes (/nl) | 1,3 | | 1,7 | 0,5 | 0,6 | 0,6 | 0,9 | 1,2 | 0,4 | 0,5 | 0,4 | 1,0 | 0,9 | 1,2 | 0,3 | 1,8 |
| Hemoglobin (g/dl) | 10,8 | | 11,0 | 10,5 | 10,2 | 10,9 | 8,2 | 9,9 | 9,6 | 7,5 | 9,0 | 10,4 | 8,8 | 12,9 | 9,1 | 8,4 |
| Platelets (/nl) | 247 | | 444 | 201 | 293 | 278 | 413 | 433 | 257 | 270 | 349 | 88 | 248 | 238 | 334 | 323 |
| Creatinine (mg/dl) | 2,0 | | 0,5 | 1,1 | 2,9 | 1,5 | 1,6 | 2,2 | 5,1 | 0,8 | 6,4 | 1,4 | 2,5 | 1,0 | 4,7 | 7,4 |
| ALT (U/l) | 29 | | 38 | 97 | 33 | 137 | 47 | 55 | 28 | 55 | 32 | 1560 | 25 | 50 | 44 | 65 |
| LDH (U/l) | 460 | | 336 | 406 | 452 | 502 | 622 | 485 | 360 | 315 | 405 | 1107 | 324 | 417 | 527 | 550 |
| CRP (mg/dl) | 297,4 | | 13,1 | 47,4 | 15,9 | 274,8 | 151,7 | 398,9 | 146,6 | 41,6 | 198,8 | 33,3 | 12,1 | 87,0 | 52,4 | 99,7 |
| PCT (ng/ml) | 3,3 | | 0,1 | 0,2 | 0,4 | 0,5 | 0,2 | 19,8 | 0,8 | 2,7 | 1,5 | 0,7 | 0,8 | 0,8 | 0,7 | 3,1 |
| Ferritin (ug/l) | 2662 | | 64 | 1607 | 755 | 1276 | 454 | 2197 | 195 | 500 | 202 | 13296 | 181 | 1187 | 185 | 752 |
| PT (quick) (%) | 58,1 | | 81,1 | 50,9 | 70,2 | 61,4 | 58,2 | 79,1 | 77,2 | 63,2 | 94,4 | 70,2 | 102,2 | 68,6 | 79,1 | 83,2 |
| D-dimer (mg/l) | 31,1 | | 11,1 | 6,2 | 9,1 | 0,9 | 11,6 | 3,5 | 4,3 | 3,1 | 4,9 | 23,0 | 3,1 | 2,5 | 2,9 | 3,9 |
| IL-6 (pg/ml) | 103,0 | | 35,4 | 25,5 | 1055,0 | 235,0 | 93,7 | 270,0 | 92,9 | 2,0 | 121,0 | 5,9 | 21,8 | 38,5 | 24,9 | 26,6 |
| pro-BNP (ng/l) | 1357 | | 1326 | 271 | 4352 | 797 | 1168 | 1131 | 6819 | 3328 | 26150 | 21619 | 10307 | 1021 | - | - |
| TNT (high sensitive) (pg/ml) | 22 | | 29 | 11 | 47 | 21 | 40 | 66 | 4020 | 22 | 857 | 377 | 39 | 19 | 175 | 121 |
| **Horowitz quotient (mmHg)** | | | | | | | | | | | | | | | | |
| - d5 |  | |  |  |  |  |  |  |  |  |  |  | 189,8 |  |  |  |
| - d10 | 197,1 | | 72,7 | 113,2 | 119,6 | - | 108,9 |  |  |  |  |  | 348,0 | 71,1 |  |  |
| - d20 |  | | 165,1 | 234,3 | 255,7 | 252,5 | 274,2 |  | 98,9 |  | 181,3 | 403,3 | 293,7 | 161,8 | 235,0 | 314,7 |
| - d35 |  | | 204,4 |  | 323,1 |  |  | 90,4 | 272,3 |  | 272,3 | 254,8 |  | 370,7 |  | 429,3 |
| **HSV-1 (copies/ml)** | | | | | | | | | | | | | | | | |
| - phase 1 |  | |  |  |  |  |  |  |  |  |  |  |  |  |  |  |
| - phase 2 | 765 | | 67000 | 165000 | 10802375 | 9888 | 6091 | 2398 | 86653 | 5807500 | 941551 | 464025000 | 42000 | 9315 | 7889 | 32505785 |
| - phase 3 |  | |  |  | 835360 |  |  | 3508 | 4370 |  | 1119812 | 266199 |  |  |  |  |
| **SARS-CoV2 (copies/ml)** | | | | | | | | | | | | | | | | |
| - phase 1 | 920000000 | |  |  |  |  |  |  |  |  |  |  | 71300000 |  |  | 22500000 |
| - phase 2 | 110000000 | | 11166 | 160800 | 321770 | 33120000 | 7820 |  | 70383500 |  | 32768333 | 2560000 | 3040500 | 6150 | 24100000 | 4946566 |
| - phase 3 |  | |  |  | 7766 | 250 |  | 15900 | 140000 |  | 3185 | 442886 |  |  |  |  |
| **Immunological findings** | | | | | | | | | | | | | | | | |
| **T-cells total (cells/ul)** |  | |  |  |  |  |  |  |  |  |  |  |  |  |  |  |
| - phase 1 | 528,0 | | 166,0 |  | 449,7 |  | 568,0 |  |  |  |  |  | 466,5 | 559,0 |  | 569,5 |
| - phase 2 | 390,5 | | 334,0 | 367,5 | 703,5 | 485,5 | 562,2 | 481,0 | 947,0 | 868,0 | 482,3 |  | 1052,8 | 938,8 | 902,5 | 1297,5 |
| - phase 3 |  | |  |  | 1283,5 | 355,0 |  | 2127,0 | 1167,0 |  | 647,0 | 706,0 | 1662,0 | 1002,3 | 1232,0 | 1217,0 |
| **CD8 total (cells/ul)** |  | |  |  |  |  |  |  |  |  |  |  |  |  |  |  |
| - phase 1 | 245,3 | | 31 |  | 110,3 |  | 128 |  |  |  |  |  | 178,5 | 94,0 |  | 88,0 |
| - phase 2 | 232,75 | | 68,0 | 127,8 | 153,5 | 246,3 | 191,4 | 167,0 | 349,0 | 247,5 | 154,5 |  | 487,0 | 184,8 | 120,0 | 379,0 |
| - phase 3 |  | |  |  | 514,5 | 160,0 |  | 894,5 | 427,0 |  | 219,5 | 297,0 | 613,0 | 299,3 | 269,0 | 288,0 |
| **CD8CD38-HLADR+ (%)** |  | |  |  |  |  |  |  |  |  |  |  |  |  |  |  |
| - phase 1 | 6 | | 14 |  | 5 |  | 23 |  |  |  |  |  | 16 | 21 |  | 18,5 |
| - phase 2 | 7,25 | | 12,0 | 30,5 | 22,3 | 55,5 | 61,8 | 13 | 12 | 39 | 32,75 |  | 39,4 | 33,9 | 29,5 | 34,7 |
| - phase 3 |  | |  |  | 67,0 | 60,5 |  | 71,5 | 13 |  | 48 | 21,5 | 43 | 41,3 | 39,0 | 10,0 |

| **Pat. No** | **15** | **3** | **6** | **1** | **13** | **14** | **12** | **4** |
| --- | --- | --- | --- | --- | --- | --- | --- | --- |
| **ISG (transkripts/1000 PPIB)** | | | | | | | | |
| **Before** | | | | | | | | |
| IL1b | 1314 | 1072 | 740 |  | 345 | 1518 | 1384 | 5291 |
| IFI44L | 104 | 208 | 47 | 54 | 152 | 188 | 1559 | 208 |
| RSAD2 | 1119 | 663 | 244 | 883 | 670 | 4265 | 3145 | 2806 |
| ISG15 | 2631 | 958 | 683 | 492 | 1605 | 7588 | 5342 | 1524 |
| MX1 | 811 | 775 | 238 | 872 | 700 | 2373 | 3234 | 558 |
| IFIT1 | 1751 | 963 | 685 | 552 | 1145 | 4617 | 5203 | 676 |
| CD8CD38-HLADR+ (%) | 15 | 5 | 23 | 4 | 21 | 12 | 17 | 4 |
| **After** | | | | | | | | |
| IL1b | 394 | 457 | 3020 | 1409 | 1632 | 9406 | 4094 | 202 |
| IFI44L | 16 | 0 | 23 | 12 | 0 | 32 | 67 | 0 |
| RSAD2 | 140 | 153 | 304 | 219 | 0 | 754 | 1526 | 0 |
| ISG15 | 725 | 621 | 368 | 300 | 607 | 2357 | 1552 | 210 |
| MX1 | 247 | 157 | 241 | 365 | 185 | 960 | 706 | 61 |
| IFIT1 | 404 | 172 | 209 | 374 | 0 | 1199 | 602 | 106 |
| CD8CD38-HLADR+ (%) | 43 | 46 | 55 | 3 | 44 | 37 | 43 | 17 |
